# Draft genome sequence of antimicrobial producing *Paenibacillus alvei* strain MP1 reveals putative novel antimicrobials

**DOI:** 10.1186/s13104-020-05124-z

**Published:** 2020-06-09

**Authors:** Magdalena Pajor, Jonathan Sogin, Randy W. Worobo, Piotr Szweda

**Affiliations:** 1 Department of Pharmaceutical Technology and Biochemistry, Faculty of Chemistry, Gdansk University of Technology, G. Narutowicza Street 11/12, 80-233 Gdańsk, Poland; 2 Department of Food Science, Cornell University, Stocking Hall, Ithaca, NY 14853-7201 USA

**Keywords:** *Paenibacillus alvei*, Whole genome sequencing, Antimicrobial-producing strain, NRPS

## Abstract

**Objective:**

A *Paenibacillus* strain isolated in previous research exhibited antimicrobial activity against relevant human pathogens including *Staphylococcus aureus* and *Listeria monocytogenes.* In this study, the genome of the aforementioned strain, designated as MP1, was shotgun sequenced. The draft genome of strain MP1 was subject to multiple genomic analyses to taxonomically characterize it and identify the genes potentially responsible for its antimicrobial activity.

**Results:**

Here we report the draft genome sequence of an antimicrobial producing *Paenibacillus* strain, MP1. Average Nucleotide Identity (ANI) analysis established strain MP1 as a new strain of the previously characterized *Paenibacillus alvei*. The genomic analysis identified several putative secondary metabolite clusters including seven Nonribosomal Peptide Synthetase clusters (NRPS) (> 10,000 nt), one bacteriocin or other unspecified Ribosomally Synthesized and Post-Translationally modified Peptide Product (RiPP), one lanthipeptide, and six hybrid clusters (NRPS-Type I Polyketide synthase (T1PKS) and NRPS-trans Amino Transferase Polyketide Synthase (AT-PKS)).

## Introduction

*Paenibacillus* spp. produce a variety of peptide and non-peptide-containing antimicrobial compounds. These compounds include hydrolytic enzymes, lantibiotics, polymyxins, paenibacterin, various organic molecules, and others [1]. Of these compounds, antimicrobial peptides are a promising group to target for the discovery of novel metabolites. These peptides are broadly classified based on their synthesis mechanism: ribosomally-synthesized bacteriocins and nonribosomally synthesized peptides (NRPs). Whereas bacteriocins are ribosomally-synthesized and post-translationally modified, NRPs are produced by large enzymes called nonribosomal peptide synthetases (NRPSs). NRPSs catalyze the incorporation and elongation of growing peptide chains without an RNA template. Regiospecific and stereospecific reactions catalyzed by NRPSs allow for the incorporation of noncanonical amino acids into these peptides, which contributes to the structural diversity of NRPs [2]. However, the discovery of NRPs is limited to genetic screens and genomic-based prediction of NRPs based on the structure of active sites in NRPSs. Therefore, many NRPs may be novel and yet to be discovered.

Although there is increasing interest in *Paenibacillus* spp. for the discovery of novel antimicrobials, the genomic information of these bacteria remains insufficient [3]. Previously, a *Paenibacillus* strain isolated from buckwheat honey [4] was identified as a producer of antimicrobial compound(s) with activity against both Gram-positive and Gram-negative pathogens, including *S. aureus* and *L. monocytogenes*. The goal of this study was to conduct whole-genome sequencing of this strain, hereafter designated MP1, to taxonomically characterize it and identify genes possibly responsible for the production of the compound(s) that yield strain MP1 its antimicrobial activity.

## Main text

### Methods

Strain MP1 was maintained at − 80 °C in 15% (vol/vol) glycerol Luria–Bertani medium (Becton–Dickinson, Franklin Lakes, NJ). Prior to extraction, strain MP1 was streaked and grown on Luria–Bertani agar. Genomic DNA was isolated from a culture of strain MP1 grown by inoculating 9 mL Luria–Bertani medium with a single colony and incubating at 37 °C for 18 h with continuous shaking at 200 rpm. DNA was extracted from 1.8 mL culture using the QiaAMP DNA Minikit (Qiagen, Germantown, MD) following a slightly modified protocol. In brief, cells were pelleted and subjected to lysozyme (20 mg/mL) treatment (Millipore Sigma, St. Lois, MO) at 37 °C for 60 min; DNA was extracted from this lysate using the QiaAMP DNA Minikit following manufacturer instructions for RNA-free genomic DNA using RNase A (Qiagen, Germantown, MD). Following extraction, DNA was spectrophotometrically quality checked using a NanoDrop (Thermo Fisher, Waltham, MA) to ensure the 260/280 and 260/230 nm absorbance ratios were greater than 1.8 and 2.0 respectively. Library preparation, quality control, and sequencing were conducted by the Cornell University Veterinary Molecular Diagnostics Laboratory utilizing the Nextera XT DNA library preparation and indexing kits (Illumina, San Diego, CA) and an Illumina MiSeq (Illumina, San Diego, CA) to obtain 2 × 250 bp paired-end reads; this yielded 3,467,242 raw reads (866 Mb).

Raw reads were trimmed and paired using Trimmomatic (v0.39) [5] with the parameters: LEADING:3 TRAILING:3 SLIDINGWINDOW:4:15 MINLEN:26. Trimmed and paired reads were first quality checked using FastQC (v0.11.8) [6] to ensure normal results for ‘per base sequence quality’, ‘per base N content’, ‘sequence duplication levels’ and ‘adapter content’, and then de novo assembled into scaffolds using SPAdes (v3.13.1) [7–9] with the parameters: -k 33,55,77,99,127 -careful. Following assembly, scaffolds less than 500 bp were removed. QUAST (v4.0) [10] was used to obtain basic assembly statistics (e.g. # scaffolds, N_50_, G + C content), and BBmap (v37.50) [11] and Samtools (v1.9) [12] were used to determine average sequencing coverage as previously described [13].

The species identification of strain MP1 was determined by ANI analysis of strain MP1′s genome versus the whole genomes of all available *Paenibacillus* type strains deposited in the National Center for Biotechnology Information (NCBI) assembly database. Following this, all-v-all ANI analysis was conducted among all strains with whole genomes deposited in the NCBI assembly database for the species of the closest related type strain. ANI analysis was conducted via the OrthoANI method using OAT (v1.40) [14] with BLAST + (v2.9.0) [15]. The species and strain names for both sets of comparisons can be found in Supplementary Data - Data 1 and Data 2. Hierarchical cluster analysis was conducted in R (v3.5.3) [16] via the complete-linkage method on dissimilarity values between strains for the species of the closest related type strain, computed as $1 - \frac{{{\text{ANI\% }}}}{100}$, to determine relatedness. Cluster analysis was visualized using R.

Genome annotation was conducted by the NCBI using the Prokaryotic Genome Annotation Pipeline (PGAP) [17, 18]. Because it was determined that strain MP1 produces antimicrobial compounds, the PGAP annotated genome was further analyzed using the AntiSMASH webserver (v5.1.0) [19] to identify putative secondary metabolite clusters and compare synteny of those clusters to related clusters in other genomes. Genes of interest identified by AntiSMASH were translated and compared to related proteins in the non-redundant protein sequences database using the NCBI BLASTp suite with default settings [20]. The genome of strain MP1 was compared to the type strain of the identified species using Mauve (v20150226) [21, 22] with progressive alignment and seed-families options, and the webserver Phaster [23, 24].

### Results

The resulting assembly of strain MP1 contained 116 scaffolds with an N_50_ value of 129,056 bp, 46.11% G + C content, and 6,511,289 bp; average sequencing coverage was 106.03 × (Additional file 1: Summary Quast Table). The draft genome of strain MP1 (annotated via PGAP) encodes 5915 genes; 5626 are coding sequences, 198 are pseudogenes, and 91 are functional or regulatory RNAs. Of the functional or regulatory RNAs, 71 are tRNAs, 5 are complete rRNAs (5–5S), 11 are partial rRNAs (1–5S, 3–16S, and 7–23S), and 4 are ncRNAs; the genome also contains 3 CRISPR arrays.

**Fig. 1:**
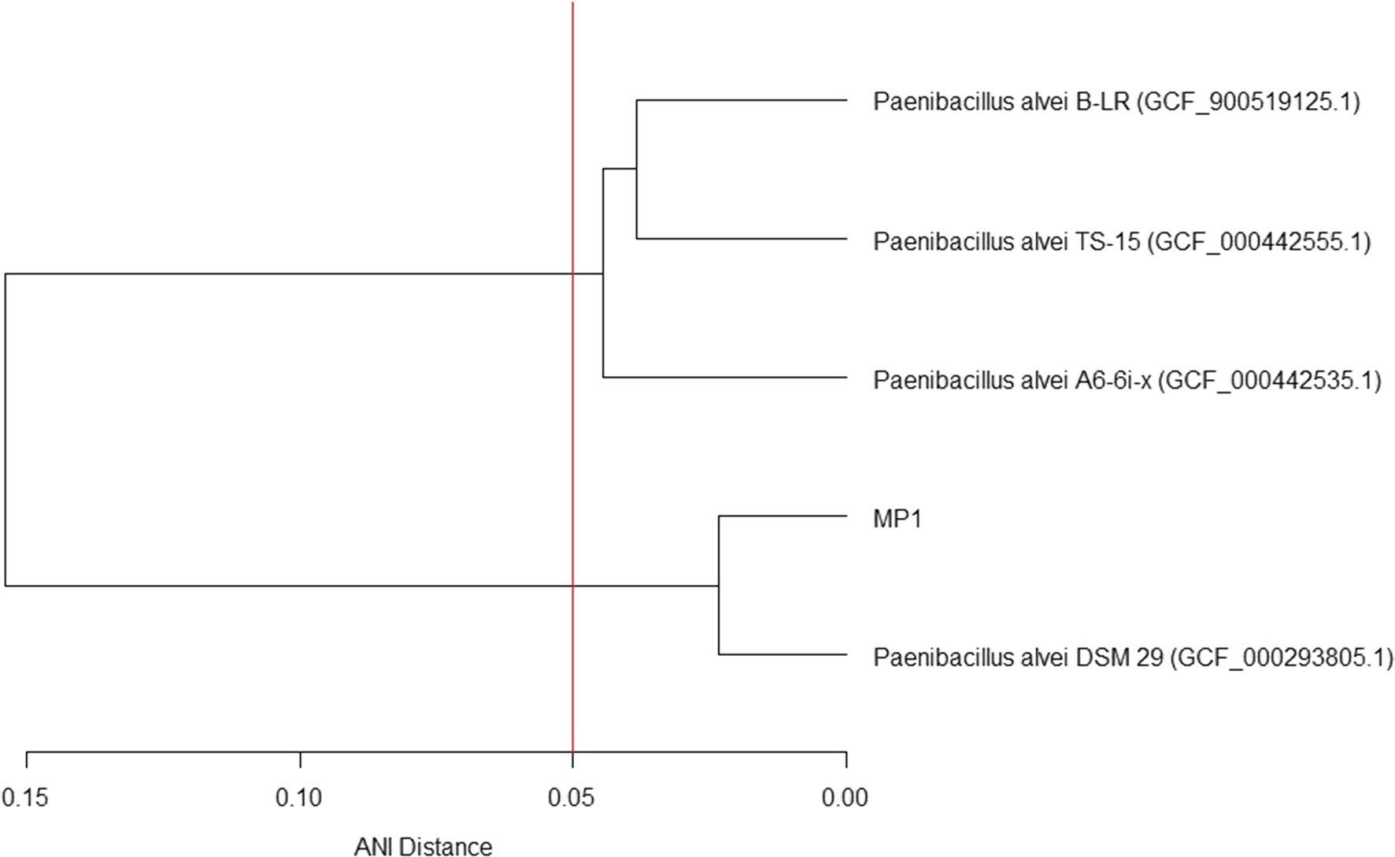
Dendrogram of ANI distances between strain MP1 and strains classified as *Paenibacillus alvei*

ANI analysis showed strain MP1 was most closely related to type strain *P. alvei* DSM 29 (97.68%, refseq accession GCF_000293805.1) (Additional file 2). When compared to other strains within the species *P. alvei*, MP1 was more distantly related: B-LR (84.89%, GCF_900519125.1), TS-15 (84.78%, GCF_000442555.1), and A6-6i-x (84.60%, GCF_000442535.1) (Additional file 3). However, these values are higher than the ANI values between strain MP1 and other *Paenibacillus* type strains (median = 67.78%, 1st quartile = 67.51%, 3rd quartile = 67.97%, n = 124). Hierarchical cluster analysis revealed two distinct clusters above 95% ANI; one cluster contained strains MP1 and DSM 29, and the other contained strains B-LR, TS-15, and A6-6i-x (Fig. 1). ANI values were computed via the orthoANI algorithm and ANI distances were calculated as 1 – ANI(%)/100. Hierarchical cluster analysis was computed via the complete-linkage method. Numbers in parentheses correspond to the NCBI RefSeq accession number for the assembly used in the analysis. The vertical red line corresponds to a 95% ANI threshold commonly used for species designations.

When comparing the genome of strain MP1 to the genome of type strain *P. alvei* DSM 29 (including 4 plasmids), subtle differences were revealed. Strain MP1 contained 626 less genes than the whole genome of type strain DSM 29 (6,541 genes). When visualized using Mauve, it became clear that divergent regions were related to the presence of mobile genetic elements (plasmids, prophage, and transposons) within each genome, specifically plasmids and prophage. This was confirmed when the individual genomes were analyzed using Phaster. The results from Phaster indicate a difference of 594 genes attributed to the presence of 13 prophage regions in strain MP1 (total = 377 genes) vs 20 in type strain DSM 29 (total = 971) (Additional files 4 and 5).

**Fig. 2:**
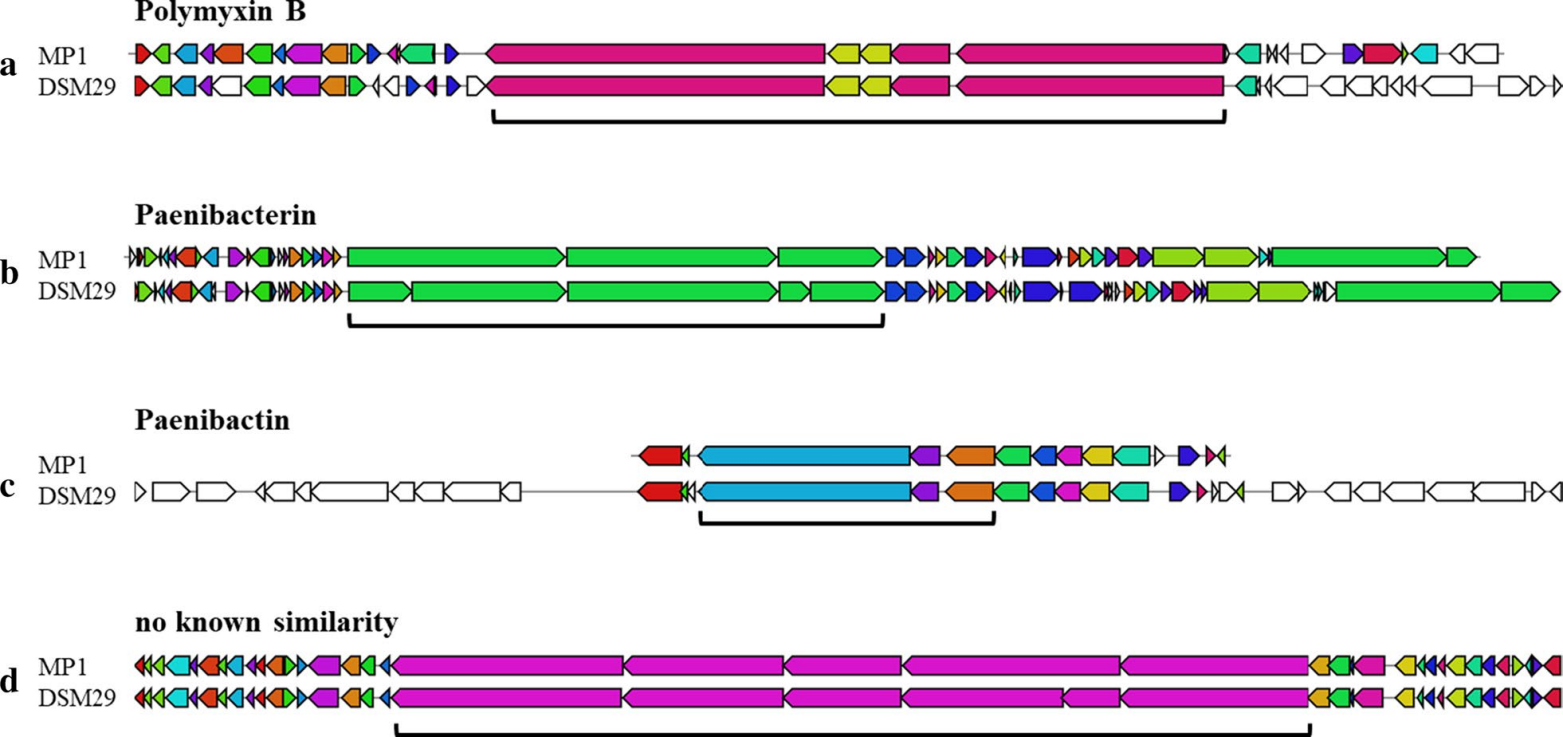
Synteny between strain MP1 and type strain DSM 29 of several NRPS clusters identified via AntiSMASH. Bracketed portions of the comparisons correspond to core biosynthetic genes for the associated NRPs

AntiSMASH analysis of the PGAP annotated MP1 genome predicted the presence of several putative secondary metabolite clusters including seven NRPS clusters (operons > 10,000 nt), one bacteriocin, one lanthipeptide, four hybrid NRPS clusters containing NRPSs and polyketide synthetases (type I or *trans*-acyltransferase), one hybrid cluster containing a lasso peptide and NRPSs, and one hybrid cluster containing a sactipeptide and resorcinol. Notably, strain MP1 contained one large operon with five putative NRPS genes spanning 71,579 nt with no similarity to genes involved in the biosynthesis of other known NRPs; type strain DSM 29 contained a similar cluster that displayed synteny with strain MP1 (Fig. 2d). In addition, other clusters encoding for a bacteriocin, sactipeptide, and lasso peptide were identified with no similarity to known antimicrobial producing gene clusters.

Three clusters were identified with 100% similarity to known NRP synthesis pathways; each of these clusters showed synteny with clusters identified in type strain DSM 29 (Fig. 2a–c). One hybrid gene cluster contained NRPS genes showing similarity to genes involved in the synthesis of polymyxin B. When the translated sequences of these genes were compared to related proteins using BLASTp, they displayed high similarity to proteins involved in the synthesis of variant forms of polymyxin B (1 and 2) produced by a strain of *P. polymyxa* [25]: PmxA (89.15%, AEZ51516.1), PmxB (96.28%, AEZ51517.1), PmxC (95.89%, AEZ51518.1), PmxD (98.61%, AEZ51519.1), PmxE (95.41%, AEZ51520.1). Another hybrid cluster contained NRPS genes showing similarity to genes involved in the synthesis of paenibacterin. When the translated sequences of these genes were compared to related proteins using BLASTp, they displayed similarity to proteins involved in the synthesis of paenibacterin produced by a strain of *P. thiaminolyticus* [26]: PbtA (67.45%, AGM16412.1), PbtB (67.70%, AGM16413.1), PbtC (60.12%, AGM16414.1), PbtD (72.87%, AGM16415.1), and PbtE (70.92%, AGM16416.1). Finally, a NRPS cluster contained NRPS genes showing similarity to genes involved in the synthesis of paenibactin. When the translated sequences of these genes were compared to related proteins using BLASTp, they displayed similarity to proteins involved in the synthesis of paenibactin produced by a strain of *P. elgii* [27]: PaeG (59.64%, AEI70240.1), PaeA (75.00%, AEI70241.1), PaeC (58.17%, AEI70242.1), PaeE (77.09%, AEI70243.1), PaeB (66.56%, AEI70244.1), and PaeF (67.78%, AEI70245.1).

### Discussion

The ANI value between MP1 and type strain *P. alvei* DSM 29 indicates that strain MP1 is a new strain within the species *Paenibacillus alvei* when considering the 95% threshold suggested by others [28, 29]. However, the ANI values between strain MP1 and other *P. alvei* strains, and the hierarchical cluster analysis, suggests that at present, *P. alvei* is a fragmented species. ANI values between type strain DSM 29 and the others were lower than 95%, which indicates those strains were originally misclassified and/or represent a novel species of *Paenibacillus*. Strains MP1 and DSM 29 both originated from honey or bee-related sources [4, 30], whereas the others did not [3, 31]; the introduction of a common ancestor to bees could have led to niche specialization that caused speciation.

The difference in the number of genes between strains MP1 and DSM 29 is almost completely attributed to differences in mobile genetic element composition, namely phage and plasmids. Type strain DSM 29 contains 4 plasmids, two which contain putative prophage; the method used to assemble the genome of strain MP1 does not attempt to identify plasmids. However, when analyzed for the presence of prophage, it became clear that almost the entire difference in the number of genes within each of the assemblies was due to differences in the composition of prophage. The remaining difference is likely due to the presence of plasmid associated maintenance genes that were not identified during prophage analysis. Varying mobile genetic element composition is reasonable to expect given the geographic origin of each isolate (MP1—Poland, DSM 29—United Kingdom); the population of phage in communities is dynamic, and geographic origin likely affected the composition of microbial communities, including phage, each isolate was exposed to. Nonetheless, due to the high ANI value between MP1 and DSM 29, strain MP1 is a new representative of the *P. alvei* species.

AntiSMASH analysis of the PGAP annotated MP1 genome confirmed its potential to produce a variety of nonribosomal peptides and polyketides. Some of the identified clusters were related to previously known compounds including polymyxin, paenibacterin, and paenibactin; others were not—such as the lasso peptide cluster. The most recent results support that the structure of paenibacterin as a cyclic lipodepsipeptide antibiotic might be useful for creating new antibiotics via synthetic routes [32], which is necessary as antimicrobial resistance arise and requires the development of novel antimicrobial agents. Furthermore, lasso peptides constitute a group of relatively new, non-toxic natural compounds with antimicrobial activity, however, their highly stable structure requires further molecular modification for potential medical applications [33]. Paeninodin synthesized by *P. dendritiformis* C454 was reported in 2016 as the novel lasso peptide tailored by a new class of kinases [34]. The potential use of these antimicrobials indicates that further investigation of antimicrobials produced by members of the genus *Paenibacillus*, including strain MP1, could lead to the discovery of medically relevant (or otherwise) compounds for use against pathogenic organisms.

Finally, this study demonstrates several antimicrobial compounds are yet to be characterized and highlights the necessity to experimentally verify the function of synthesis genes. Such efforts will result in a better understanding of the structure and synthesis of antimicrobial compounds and will lead to better genome-based predictions in the future.

## Limitations

The acknowledged analysis is based on the draft genome of strain MP1. Therefore, regions of the complete genome may be duplicated or missing from the assembly.

## Supplementary information

**Additional file 1: Data 1.** The QUAST Summary table.

**Additional file 2: Data 2.** OrthoAni_Paenibacillus_type_strains.

**Additional file 3: Data 3**. OrthoAni_Paenibacillus_alvei.

**Additional file 4: Data 4**. DSM29_prophage_supplement.

**Additional file 5: Data 5.** MP1_prophage_supplement.

## Data Availability

This Whole Genome Shotgun project has been deposited at DDBJ/ENA/GenBank under the accession WSQB00000000. The version described in this paper is version WSQB01000000. The *P.alvei MP1* strain is available upon request from R.W. Worobo or P. Szweda.

## Abbreviations

ANI: Average Nucleotide Identity
NRPS: Nonribosomal Peptide Synthetase clusters
NRPSs: Nonribosomal Peptide Synthetases
RiPP: Ribosomally Synthesized and Post-translationally modified Peptide Product
T1PKS: Type I Polyketide synthase
AT-PKS: Amino Transferase Polyketide Synthase
NCBI: The National Center for Biotechnology Information
PGAP: Prokaryotic Genome Annotation Pipeline
SRA: Sequence Read Archive
Nt: Nucleotide
