# Supplementary material for: Draft genome sequence of antimicrobial producing Paenibacillus alvei strain MP1 reveals putative novel antimicrobials
Source: BMC Res Notes. 2020 Jun 9;13:280. doi: 10.1186/s13104-020-05124-z (PMC7285544; doi:10.1186/s13104-020-05124-z)
Supplement: Supplementary file 1 — Additional file 1: Data 1. The QUAST Summary table. [file 13104_2020_5124_MOESM1_ESM.docx]

Table 1 The QUAST summary table

| Metric | Value |
| --- | --- |
| # contigs 1 – 999 (bp) | 19 |
| # contigs 1,000 – 4,999 (bp) | 22 |
| # contigs 5,000 – 9,999 (bp) | 8 |
| # contigs 10,000 – 24,999 (bp) | 13 |
| # contigs 25,000 – 49,999 (bp) | 11 |
| # contigs ≥ 50,000 (bp) | 43 |
| Total # contigs | 116 |
| Smallest Contig (bp) | 560 |
| Largest Contig (bp) | 484,246 |
| Total Length (bp) | 6,511,289 |
| GC (%) | 46.11 |
| N50 (bp) | 129,056 |
| L50 (# contigs) | 14 |
| # Ns (per 100 kbp) | 3.01 |
